# The Vaginal *Lactobacillus gasseri* YS1021 Effectively Degraded Mucin by Enzymolysis of M13 Metallopeptidase

**DOI:** 10.3390/microorganisms14051136

**Published:** 2026-05-17

**Authors:** Jialing Hou, Jingyu Miao, Yanyan Dai, Yu Cui, Sheng Yin, Yunbo Luo

**Affiliations:** 1 School of Food & Health, Beijing Technology & Business University, Beijing 100048, China; hjl000109@163.com (J.H.); 15037402201@163.com (J.M.); dyanyan227@163.com (Y.D.); 2 State Key Laboratory of Medical Proteomics, National Center for Protein Sciences (Beijing), Beijing Institute of Lifeomics, Beijing 102206, China; cuiyubmi@163.com; 3 BTBU-CAU Joint Laboratory of Synthetic Biology for Food, Nutrition and Health, Beijing Technology & Business University, Beijing 100048, China

**Keywords:** vaginal lactobacilli, *Lactobacillus gasseri*, mucin degradation, metallopeptidase, nutrient acquisition

## Abstract

Mucin is the core component of the mucus layer barrier in the human reproductive tract and the interaction between mucin and commensal bacteria is crucial for the female reproductive health. In this study, we found that the female reproductive tract isolate *Lactobacillus gasseri* YS1021 could degrade and utilize mucin to obtain the promoted proliferation. After 48 h of cultivation with 1% mucin added, the biomass of strain YS1021 increased fourfold compared to the control group without mucin. Meanwhile, the addition of mucin induced a 5-fold higher expression of the M13 family metalloprotease encoding gene *RS7445*, revealing that the enzyme was associated with mucin degradation in the strain YS1201. Moreover, the recombinant strain *L. gasseri* 7445OE with *RS7445* over-expression exhibited an enhanced mucin degradation ability and reached a 7-fold higher biomass than the parent strain YS1021 after 48 h of cultivation with addition of 1% mucin. Furthermore, the gene *RS7445* was expressed in *Escherichia coli* BL21, and the 74 kDa recombinant protein of RS7445 was successfully purified. Enzymatic hydrolysis assay in vitro showed that mucin was decomposed into 39 peptide fragments by the recombinant RS7445, which demonstrated that the M13 family metalloprotease contributes to mucin degradation in *L. gasseri* YS1021. In sum, this study revealed an important mechanism by which the vaginal *L. gasseri* YS1021 could degrade and utilize mucin to gain a growth advantage.

## 1. Introduction

The human reproductive tract is covered by the mucus layer, which acts as a natural defense barrier that physically isolates pathogens and prevents epithelial cells from pathogens invasion [1]. The mucus is composed of water, mucin, electrolytes, and non-specific antibacterial proteins (IgA, lactoferrin, and lysozyme) [2,3]. Mucin is the core structural component of the mucus layer and it is characterized as a glycoprotein polymer composed of peptides rich in serine and threonine residues and oligosaccharides attached to the peptide chain [4]. Mucin plays an indispensable role in the interaction between bacterial community and the mucus layer in the reproductive tract. On one hand, mucin generated from the regularly renewed mucus layer could provide nutrients for commensal bacteria; on the other hand, pathogenic bacteria could degrade mucin, destroy the intact structure of the mucus layer, and consequently directly invade epithelial cells and cause bacterial vaginosis (BV) [5]. It’s reported that the vaginal pathogen *Gardnerella vaginalis* could secrete sialidase to hydrolyze mucin on the mucosa and evade the host’s immune attack. The pathogen could also produce vaginal hemolysin, which dissolves vaginal epithelial cells and exacerbating the clinical symptoms of BV [6]. However, there are few reports on degradation and utilization of mucin by the reproductive tract probiotics. But attentions have been focused on the interaction between human intestinal microbiota and mucin in the intestinal mucus layer. Ruas-Madiedo et al. reported two bifidobacteria isolates could degrade intestinal mucin by two extracellular glycosidases (AfcA and EngBF) [7]. In addition to the glycosidase hydrolyzing oligosaccharide chain, metal peptidases play important roles in mucin degradation. The M66 family metalloprotease StcE of enterohaemorrhagic *Escherichia coli* could reduce the inner mucus layer of human colon mucosal biopsy and the MUC2 glycoprotein level in the mucus producing LS174T colon cancer cells [8]. The protease Pic of *Shigella flexneri* and enteroaggregative *E. coli* exhibited proteolytic activities on bovine and mouse mucin [9]. Apparently, the oligosaccharide chain hydrolyzing glycosidase and the peptide chain decomposing protease are involved in microbial mucin degradation and utilization.

Strains belonging to the species *Lactobacillus gasseri* are some of the dominant probiotics in the female vagina [10]. Genome analysis indicated that *L. gasseri* contained abundant putative mucin-binding proteins, making it have the possible advantage in effective adhesion to the mucin layer [11]. But it is generally acknowledged that *L. gasseri* was deficient in sugar hydrolases required for the utilization of complex carbohydrates. Due to the complex oligosaccharide chains attached to the peptide chain of the vaginal mucin, it’s traditionally believed that *L. gasseri* and other probiotics lacking glycosidases could hardly effectively utilize mucin in the reproductive tract [12]. However, some studies reported that the *L. gasseri* strains in the intestinal tract possessed diverse PTS transport proteins and sugar hydrolases for monosaccharides and disaccharides, which enables it to gain a competitive advantage in the digestive tract [13]. With respect to *L. gasseri*, few studies have been reported on how it interacts with mucin in the reproductive tract. In this study, the *L. gasseri* strain YS1021 isolated from female vaginal samples was identified to have a capacity of effective utilization of mucin to obtain a growth advantage. Moreover, the M13 family metallopeptidase RS7445 was in vitro verified to degrade mucin into peptide fragments. The results revealed a possible mechanism that the vaginal *L. gasseri YS1021* could obtain an advantage of survival competition by mucin degradation and utilization.

## 2. Materials and Methods

### 2.1. Strains, Plasmids and Culture Conditions

Table 1 indicates the plasmids and bacterial strains used in this study. Anaerobic conditions were used to cultivate *L. gasseri* strains in MRS medium at 37 °C. *E. coli* strains were cultivated at 37 °C with vigorous shaking in LB medium. Final antibiotic concentrations for selection, when needed, were as follows: 5 μg/mL erythromycin for *L. gasseri*, and 50 μg/mL kanamycin or 500 μg/mL erythromycin for *E. coli*.

To evaluate the influence of mucin on bacterial growth, fresh cultures of *L. gasseri* strains were inoculated (2%, *v*/*v*) into B medium (0.04% urea, 0.1% acetic acid, 0.14% potassium hydroxide, 0.02% calcium hydroxide, 0.02% glycerol, 0.2% lactic acid, 0.35% sodium chloride, 0.5% glucose, pH 7.0), B+M medium (1% mucin (CAS No. 84082-64-4, Sigma, St. Louis, MO, USA) was added into B medium, pH 7.0), or B+N medium (0.6% peptone, 0.2% beef extract, and 0.2% yeast extract were added into B medium, pH 7.0), respectively. The commercia mucin was treated with purification and freeze-drying before use for media preparation. Cell growth was measured by absorbance at OD_600_ nm after 48 h of anaerobic cultivation at 37 °C.

### 2.2. DNA Manipulation Techniques

According to Green and Sambrook (2012), standard DNA manipulation procedures were carried out [14]. According to the manufacturer’s instructions, the bacterial DNA kit and plasmid purification kit were used to prepare genomic DNA and plasmid DNA (TransGen Biotech, Beijing, China). The TaKaRa primers SRTAR MAX DNA polymerase was used for DNA amplification (TaKaRa, Kusatsu, Japan). DNA ligation and restriction enzyme digestion were performed following the supplier’s instructions (TaKaRa). Plasmid DNA was introduced to *E. coli* using the conventional heat shock transformation procedure [14]. With a few adjustments, electroporation was utilized to transform *L. gasseri* [15]. Briefly, an ice-cold electroporation cuvette was filled with 100 μL of competent cells along with 1.0 μg of pure DNA. The electric pulse was then delivered using the following parameter settings: 2.0 kV, 25 μF, and 400 Ω. Following the electric shock, 1 milliliter of the recovery medium (MRS supplemented with 0.1 M MgCl_2_ and 0.3 M sucrose) was added right away. The recovery culture was typically incubated in anaerobic conditions for three hours at 37 °C. Total mRNA was extracted using the Trizol extraction kit (TransGen Biotech, Beijing, China), and then RNA was reverse-transcribed using the FastKing reverse transcription kit (TransGen Biotech, Beijing, China) to generate cDNA. RT-qPCR was performed using the NovoStart SYBR Green Color qPCR Supermix Kit (TransGen Biotech, Beijing, China) on the CFX96 system (Bio-Rad, Hercules, CA, USA). The cycling conditions were as follows: 95 °C for 30 s, then 45 cycles consisting of 94 °C for 5 s, 56 °C for 15 s, and 72 °C for 25 s. Normalization of transcript levels was achieved using the *GADPH* gene. Data were then analyzed by the 2^−^^ΔΔCt^ method [16]. The primers employed in this study are shown in Table 2.

### 2.3. Construction of the Recombinant Strain L. gasseri 7445OE with Over-Expression of the Metallopeptidase Gene RS7445

Using primers P1/P2, PCR amplification was performed to obtain the gene *RS7445* from the genomic DNA of *L. gasseri* YS1021. The gene *RS7445* was digested by *Nco* I and *Eco*R I, and added into the *Lactobacillus* expression vector pSIP411 by ElectroLigase (TaKaRa). The ligation product (pSIP7445) was electroporated into *L. gasseri* YS1021, and transformants were selected on MRS agar plates containing erythromycin [17,18]. Plasmid profiles and sequencing analysis using the NCBI’s BLAST Program against the GenBank database (URL: https://blast.ncbi.nlm.nih.gov/Blast.cgi.) were used to verify the recombinant *L. gasseri* 7445OE.

### 2.4. Evaluation of Mucin Degradation by the Recombinant Strain L. gasseri 7445OE

To evaluate the effect of the gene *RS7445* over-expression on bacterial growth with addition of mucin, the recombinant strain *L. gasseri* 7445OE and its parent wild strain *L. gasseri* YS1021 were inoculated (2%, *v*/*v*) into media B, B+M, and B+N, respectively. Cells were incubated in 96-well microplates at 37 °C for 48 h under anaerobic conditions [19]. Microbial growth was monitored by Microorganism Growth Curve Monitoring System of microplate reader (BMG LABTECH, Ortenberg, Hesse, Germany). The content of mucin left in the B+M media was determined every 8 h during bacteria cultivation using the MUC2 ELISA Kit (Fine Biotech, Wuhan, China).

To evaluate the capacity of mucin degradation, the recombinant strain *L. gasseri* 7445OE and the wild strain *L. gasseri* YS1021 were inoculated (2%, *v*/*v*) into the agar plates of media B, B+M, and B+N, respectively. After anaerobic cultivation at 37 °C for 72 h, the agar plates were staining in acetic acid solution (3.5 M) containing 0.1% amino black 10B for 30 min and washed clean using acetic acid solution (1.2 M). The capacity of mucin degradation was defined by the size of transparent dissolution zones that appeared on the agar plates.

### 2.5. Induced Expression and Purification of the Metallopeptidase RS7445 in E. coli

The amplicons of the metallopeptidase gene *RS7445* using primers P7/P8 and the linear vector pET-28a were fused into the recombinant expression vector pET7445 using the Seamless Cloning and Assembly Kit (TaKaRa). After transforming the pET7445 vector into *E. coli* TOP10, we selected transformants on LB agar plates containing kanamycin. The vector pET7445 was verified through sequencing and sequence comparison.

Transformation of *E. coli* BL21(DE3) with the pET7445 vector produced the recombinant strain *E. coli* 7445OE, allowing for induced expression of RS7445. The overnight culture of *E. coli* 7445OE (1%, *v*/*v*) was inoculated into 20 mL of fresh LB media containing kanamycin and cultivated at 37 °C with vigorous shaking at 200 rpm. When OD_600_ reached 0.5, isopropyl-β-d-thiogalactoside (IPTG) was added into the culture at the final concentration of 0.5 mM. The culture was then cultivated at 30 °C with vigorous shaking at 150 rpm for 12 h. Cells were collected by centrifuge at 4 °C at 6000 rpm for 10 min and washed clean using PBS buffer solution. To obtain cell lysates, samples were sonicated in an ice bath for 10 min using a SCIENTZ JY88-IIN sonicator (Ningbo, China, 225 W output) with alternating 6 s pulses and 3 s intervals. After ultrasonic treatment, low-temperature centrifugation separated the cell debris from the clear supernatant. The collected samples were then separated by SDS-PAGE on a 12% polyacrylamide gel, and the gel was subsequently scanned with a BioSpectrum Imaging System (Bio-Rad). The recombinant protein content was derived from the OD value as described in the reference [20].

The purification of recombinant protein RS7445 was initially carried out by filtering the cell lysate through a 0.22-micron membrane. The filtrate was then loaded onto a Ni column containing Ni-NTA resin (TRANSGEN, Beijing, China) as per the supplier’s instructions. The 6×His-tagged RS7445 protein was eluted. The eluate was collected and analyzed by SDS-PAGE.

The enzymatic activity of recombinant RS7445 was detected by collagenase spectrum method [21]. The active protein of RS7445 was prepared using conventional methods without addition of EDTA or β-mercaptoethanol. A 10% acrylamide gel containing collagen was prepared for electrophoresis. The completely denatured sample (100 °C, 3 min) and the active sample of RS7445 were used for electrophoresis at 150 V. After protein bands were separated well in electrophoresis, the gel was incubated with the enzymatic renaturation buffer (50 mM Tris, 10 mM CaCl_2_, 50 mM NaCl, 0.05% Brij-35, 1 μM ZnCl_2_, pH 7.5) at 37 °C for 48 h, followed by Commassie Blue staining and decolorization. The enzymatic activity of RS7445 was indicated by formation of light-colored bands of collagen by enzymolysis of renatured RS7445 in the gel.

### 2.6. Analysis of Enzymatic Hydrolysis Products of Mucin by the Metallopeptidase RS7445

Mucin (10 mg/mL) was digested by the recombinant RS7445 (30 mg/mL) in the reaction buffer (50 mM Tris-HCl, 0.2 M NaCl, 5 mM CaCl_2_, 1 μM ZnCl_2_, pH 7.5) at 37 °C for 48 h and used for enzymatic hydrolysis products analysis by LC-MS/MS. The same amount of mucin that was incubated with inactivated RS7445 in the same condition was used as the control sample. LC-MS/MS analysis was performed using a nanoflow LC system (EASY-nLC 1200, Thermo Fisher Scientific) connected online to a Q Exactive HF Hybrid Quadrupole-Orbitrap mass spectrometer (Thermo Fisher Scientific, Waltham, MA, USA). Peptides were first loaded onto a 1 cm self-packed trap column (150 μm inner diameter, 1.9 μm resin, ReproSil-Pur C18-AQ, Dr Maisch GmbH) in solvent A (0.1% formic acid, FA, in HPLC-grade water). Following loading and washing, the retained peptides were transferred to a 15 cm separation column (same resin and inner diameter) and resolved over a 75 min non-linear gradient from 6% to 95% solvent B (0.1% FA in ACN) at a flow rate of 600 nL/min. Ionization of the peptides employed a 2.0 kV spray voltage and a capillary temperature of 320 °C, and data were acquired in OT-OT mode. Full MS scans (300–1400 *m*/*z*) were collected at 120,000 resolution, using a 50 ms maximum injection time and an AGC target of 3e6. MS/MS spectra were generated by HCD (27% normalized collision energy) for up to 30 precursors, with a 15 s dynamic exclusion window. The Orbitrap read out the resulting MS2 spectra at 15,000 resolution, an AGC target of 5 × 10^4^, and a 20 ms injection time. The acquired MS/MS spectra were searched by Mascot 2.3 (Matrix Science Inc.) against the mucin database (https://www.medkem.gu.se/mucinbiology/databases/db/Mucin-pig-2015.htm, accessed on 20 March 2026). The protein sequence of mucin from pig was used for peptide search and blast. The identified peptides with a Mascot score higher than 10 were selected and retained as high-credibility results. The parameter settings were as follows: the mass tolerances were defined as 20 ppm for precursor ions and 50 mmu for product ions. A maximum of two missed cleavages was permitted. Oxidation of methionine (M) was specified as a dynamic modification.

## 3. Results

### 3.1. L. gasseri YS1021 Could Utilize Mucin as a Nitrogen Source Nutrient

**Figure 1:**
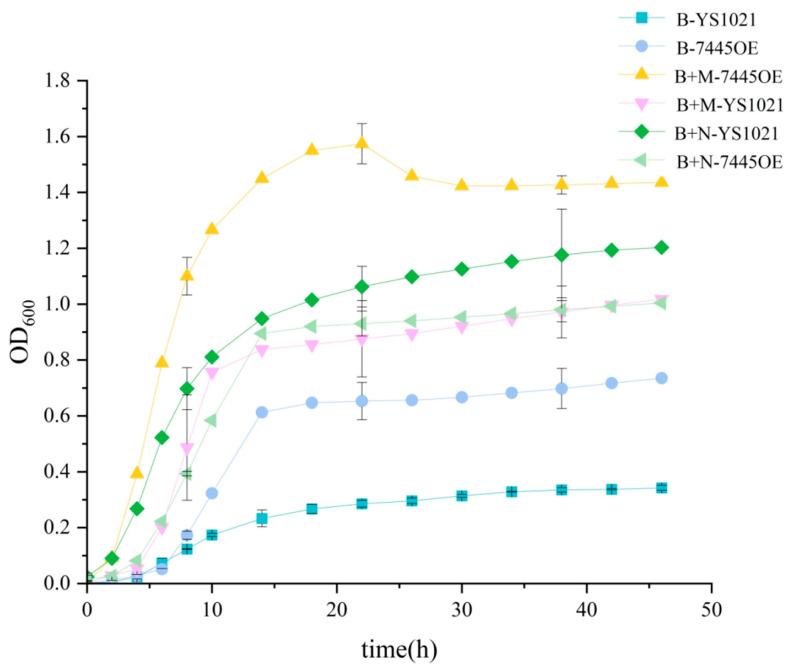
Growth curves of *L. gasseri* strain YS1021 and 7445OE cultured in media B, B+N, and B+M. Data represent the mean ± SD of three biological replicates.

The chemical defined media (B) which approximately simulates the reproductive tract environment was used to evaluate the capacity of mucin utilization by *L. gasseri* YS1021. As shown in Figure 1, when cultured in the media B, the strain YS1021 hardly grew well due to lack of nitrogen source compounds. In contrast, when conventional nitrogen sources (N) or mucin (M) was added into the media B, the biomass of YS1021 increased rapidly during 48 h of cultivation. Though the biomass of YS1021 in presence of conventional nitrogen sources was relatively higher, addition of mucin compensated for lack of nutrition and consequently helped the strain achieve a normal growth. The results suggested that mucin probably could be utilized as a nitrogen source by *L. gasseri* YS1021.

### 3.2. The Metallopeptidase Gene RS7445 Was Expressed at a High Level in Response to Mucin Degradation by L. gasseri YS1021

**Figure 2:**
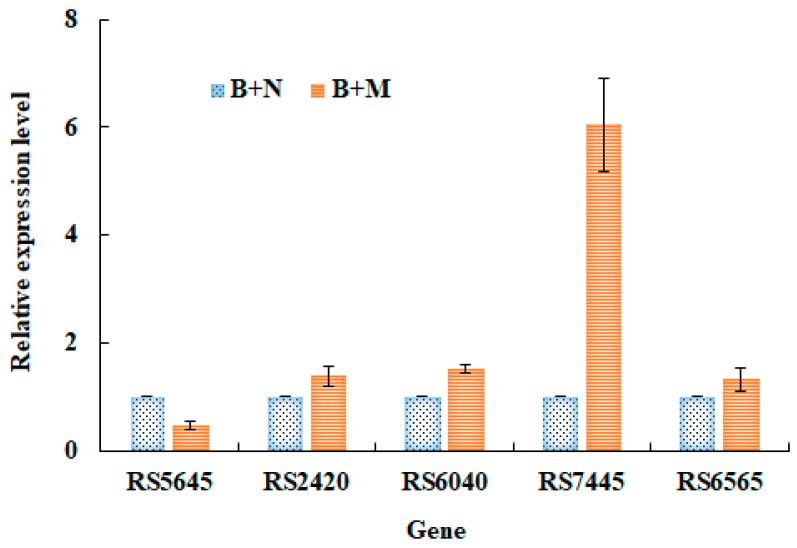
Expression levels of five peptidase genes in *L. gasseri* YS1021 cultured in media B+N and B+M. Data represent the mean ± SD of three biological replicates.

In order to identify the key enzyme responsible for mucin utilization, the cultures of *L. gasseri* YS1021 with and without addition of mucin were subject to transcriptome analysis . Five peptidase encoding genes were identified to possibly get involved in mucin utilization according to their up-regulated expression levels in response to the presence of mucin (Table 3). The gene *RS7445* exhibited the highest expression level among the five candidate genes. Furthermore, the RT-qPCR assay was performed to verify the expression levels of five peptidase genes under the conditions of bacterial cultivation with addition of conventional nitrogen sources (N) and mucin (M). Particularly, only the gene *RS7445* showed a significant 6-fold higher expression in response to the culture with addition of M than that with addition of N in the media, while other genes did not show significantly differential expressions under the same cultivation conditions (Figure 2). The results indicated that the presence of mucin remarkably induced the gene *RS7445* expression and the inducible expression was hardly interfered by the presence of other conventional nitrogen source compounds in the media. Sequence analysis showed that the *RS7445* gene was 1947 bp in size and encoded 648 amino acid residues. Its protein sequence shared a 100% similarity with the M13 family metalloprotease of *L. gasseri* (GenBank Accession No. WP_095670228.1). It’s reasonable to suspect that the M13 metalloprotease RS7445 was strongly associated with mucin degradation by *L. gasseri* YS1021.

### 3.3. Over-Expression of the Metallopeptidase Gene RS7445 Enhanced the Capacity of Mucin Degradation of L. gasseri YS1021

The metallopeptidase gene *RS7445* was over-expressed in *L. gasseri* YS1021 to evaluate its role in mucin degradation. The *RS7445* gene cloned from *L. gasseri* YS1021 was inserted into the vector pSIP411, and was transformed into *L. gasseri* YS1021, generating the recombinant strain *L. gasseri* 7445OE. RT-qPCR analysis revealed that the *RS7445* transcript level in the recombinant strain 7445OE was 3.8-fold higher than that observed in the parent strain YS1021. Bacterial growth curves showed that the recombinant strain 7445OE cultured in the media B+M obtained the most advantageous growing status (Figure 1). When cultured in the media B+N, the recombinant strain 7445OE displayed a less vigorous growth than the parent strain YS1021. As for the parent strain YS1021, the biomass cultured in the media B+N was higher than that in the media B+M. Obviously, over-expression of the metallopeptidase gene *RS7445* endowed *L. gasseri* YS1021 with an intensified capacity of mucin utilization, which consequently helped the host obtain a growth advantage.

**Figure 3:**
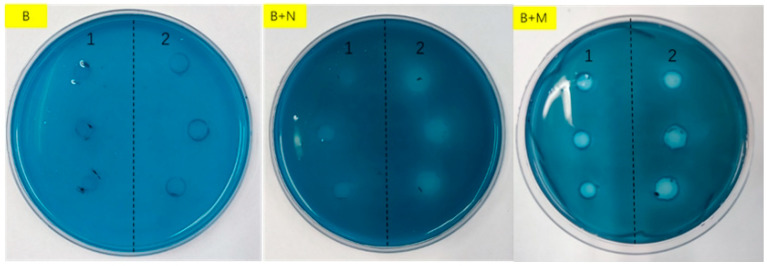
Cultivation of *L. gasseri* strain YS1021 (1) and 7445OE (2) on mucin degradation agar plates of media B, B+N, and B+M.

**Figure 4:**
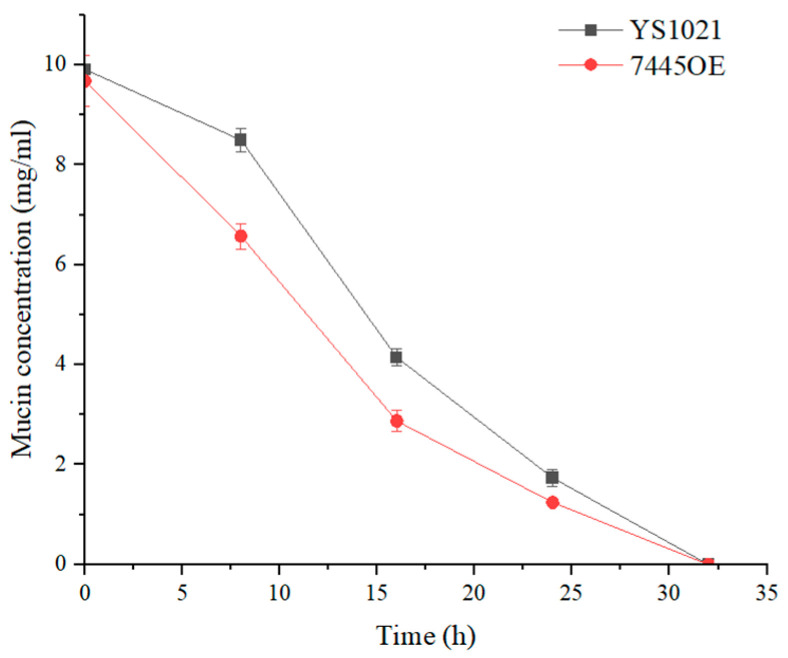
Determination of mucin content in the media B+M inoculated with *L. gasseri* strain YS1021 and 7445OE. Data represent the mean ± SD of three biological replicates.

The mucin degradation agar plates were used to evaluate the degree of mucin degradation in the media by *L. gasseri* YS1021 and 7445OE. As shown in Figure 3, the transparent dissolution zones that indicated mucin degradation appeared around colonies *L. gasseri* YS1021 and 7445OE on the B+M agar plates. The transparent dissolution zones formed by the recombinant strain 7445OE was about two times bigger in size than that by the parent strain YS1021, which indicated that the strain 7445OE possessed a stronger ability of mucin degradation. Moreover, the mucin consumption by *L. gasseri* YS1021 and 7445OE was determined using an ELISA method. During 32 h of cultivation in the media B+M, mucin was consumed faster by the strain 7445OE than that by the strain YS1021 (Figure 4).

### 3.4. Expression and Purification of the Metallopeptidase Gene RS7445 in E. coli and Analysis of Enzymolysis Products of Mucin by the Purified RS7445

**Figure 5:**
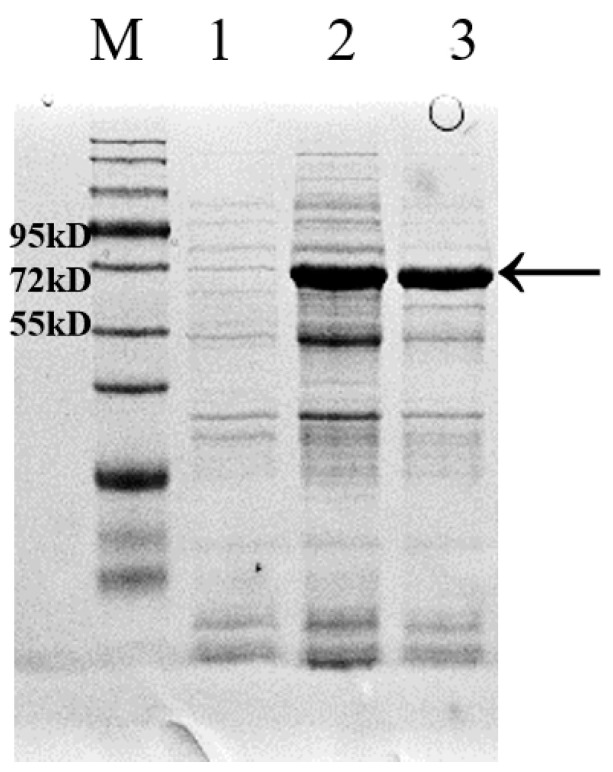
SDS-PAGE analysis of protein samples from *E. coli* 7445OE. M, protein marker; 1, the protein sample of cell lysate without IPTG induction; 2, the supernatant sample of cell lysate with IPTG induction; 3, the sediment sample of cell lysate with IPTG induction; RS7445 was indicated by the arrow.

**Figure 6:**
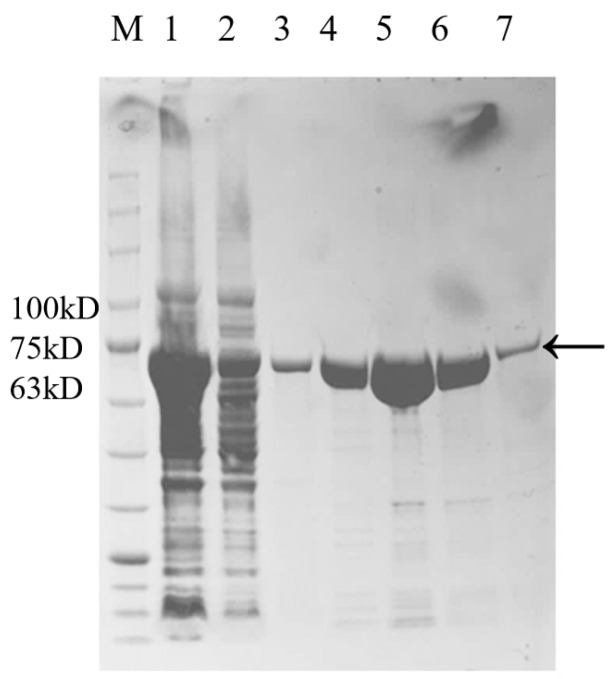
SDS-PAGE analysis of purified RS7445. M: protein marker; 1, protein sample without purification; 2, protein sample of flow-through; 3~7: protein sample of eluted sample with 50 mM, 100 mM, 150 mM, 200 mM, and 250 mM imidazole eluents; RS7445 was indicated by the arrow.

**Figure 7:**
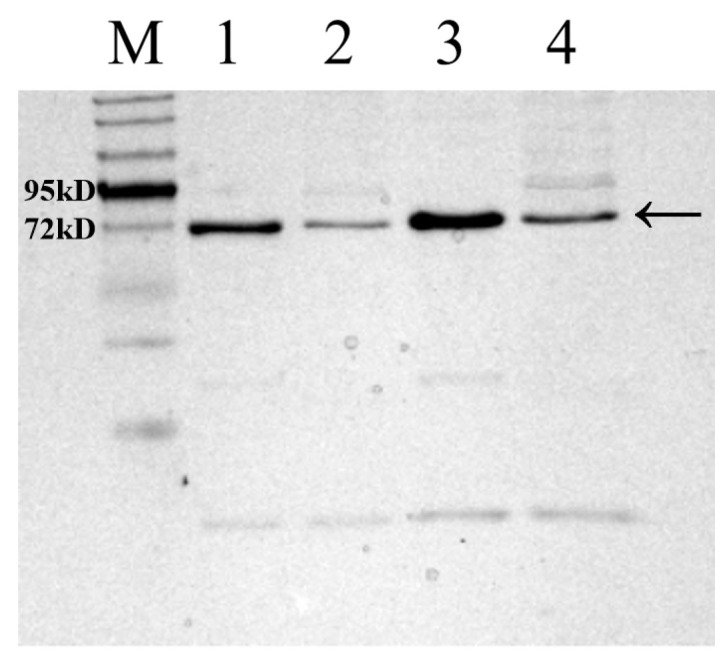
SDS-PAGE analysis of collagen by enzymolysis of denatured and renatured RS7445. M: Protein marker; 1 and 3, collagen bands incubated with completely denatured RS7445; 2 and 4, collagen bands incubated with renatured RS7445; RS7445 was indicated by the arrow.

**Figure 8:**
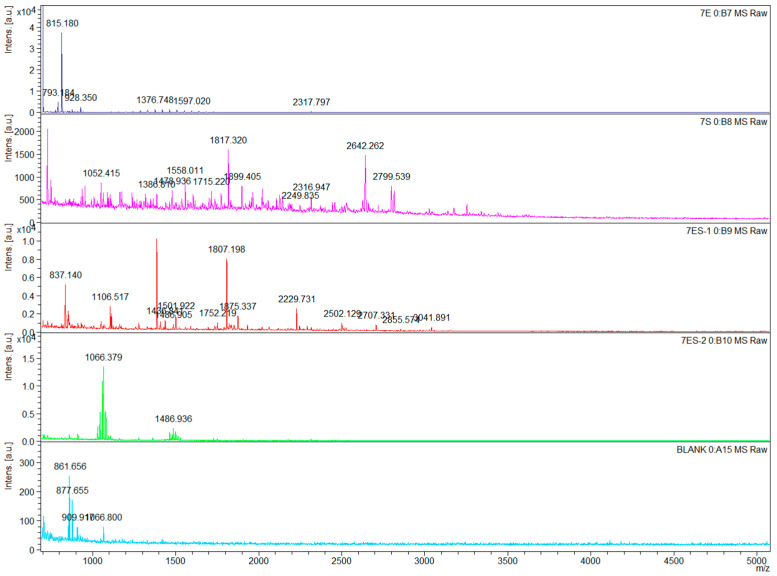
Secondary mass spectrum of the enzymolysis products of mucin by the purified RS7445. BLANK, the control sample of mucin incubated with inactivated RS7445.

The *RS7445* gene cloned from *L. gasseri* YS1021 was fused with the inducible expression vector pET-28a, and the recombinant expression vector pET7445 was transformed into *E. coli* BL21 (DE3), generating the recombinant strain *E. coli* 7445OE for induced expression of *RS7445*. As shown in Figure 5, in comparison with the protein sample without induced expression, the recombinant 74 KDa RS7445 was accumulated and detected in both the supernatant and the sediment of cell lysate of the strain *E. coli* 7445OE under the cultivation condition with IPTG induction. The recombinant RS7445 was subsequently purified on a Ni column packed with Ni^2+^-chelated beads, followed by elution with imidazole buffers ranging from 50 mM to 250 mM (Figure 6). The purified protein of recombinant RS7445 (0.7 mg/mL) was prepared by the 250 mM imidazole eluent for subsequent enzymatic activity assay and enzymolysis products analysis. The enzymatic activity of the purified RS7445 was assayed by the collagen enzymolysis method. As shown in Figure 7, light-colored protein bands of collagen were observed in the electrophoresis lane of renatured RS7445, while more stained protein bands of collagen were detected in the contrast lane of completely denatured RS7445. It’s demonstrated that the active protein of RS7445 could degrade collagen to a certain degree. In order to further verify the functionality of RS7445, mucin was incubated with the purified RS7445 and the enzymolysis products were analyzed by LC-MS/MS. The mass spectrum indicated that 39 peptide fragments were detected in the enzymolysis products of mucin (Figure 8), which proved that the metallopeptidase RS7445 could directly degrade mucin into peptide fragments in vitro. The mucin peptides selected by high credibility were shown in the Supplementary File.

## 4. Discussion

Mucin degradation and utilization is an important way to obtain nutrients for microbial flora in the human ecological niches including the genital tract and the intestinal tract, which is also one driven factor that could shape the microbial community dynamics. In the intestinal tract, both probiotics (*Akkermansia muciniphlia*, *Bifidobacterium*) and pathogens (*E. coli*, *Shigella*) are reported to have the ability to degrade and utilize mucin and consequently exert positive or negative effects on mucosal homeostasis [7,8,9,22,23]. As for the female genital tract, the traditional view holds that pathogens such as *Gardnerella* could break down mucin and the mucus layer by secretion of sialidases, glycosidases, and proteases [6,24,25], but it’s believed that the commensal *Lactobacillus* species such as *L. gasseri* could hardly utilize mucin due to deficiency in sialidases and glycosidases [12]. However, our study confirmed that vaginal lactobacilli can effectively break down mucin, which would probably overturn the obsolescent perception mentioned above. In our study, 15 vaginal lactobacilli strains including *L. gasseri*, *Lactobacillus crispatus*, *Lactobacillus jensenii*, *Lactiplantibacillus plantarum*, and *Limosilactobacillus fermentum* were used for mucin degradation assay and the species *L. gasseri*, *L. crispatus*, and *L. jensenii* were proved to have the capacity of mucin consumption. The diversity in the capacity of mucin degradation among these vaginal lactobacilli suggests the possibility of differential competitive advantage in nutrition acquisition in the genital tract, where mucin may serve as a major source of nitrogen. However, a hypothetical concern may be raised that mucin-degradation *Lactobacillus* strains would possibly exert destructive effects on mucus layer integrity like pathogens of *Gardnerella* in the genital tract. So far, no solid evidence is observed to conclude mucin-degradation lactobacilli strains could play probiotic or virulent roles in the vaginal niche. Therefore, in-deep studies are indispensable to provide fundamental experimental evidences using animal models or clinically relevant host-associated models under biologically representative conditions, which will help reveal the ecological influence of mucin-degradation lactobacilli strains on mucus layer. In this work, it’s confirmed that the vaginal *L. gasseri* YS1021 can effectively degrade and utilize mucin to gain a growth advantage, at least implying that mucin degradation and utilization would be a beneficial characteristic for survival of the strain. This study focused on figuring out how the vaginal *L. gasseri* YS1021 interacted with mucin, which would help us understand the survival strategy of *L. gasseri* in the female genital tract.

The degradation of mucin has been studied in prominent members of the human gut microbiota. A putative model has been proposed that mucin decomposition needs a synergistic involvement of glycosidase, sulfatase, sialidase and proteolytic peptides [26]. The exo-acting glycosidases are expressed to initiate mucin breakdown, which are responsible for extracellular sequential trimming of terminal sugars from the O-glycan side chains, eventually results in exposing the peptide backbone for proteolysis [27]. In contrast, different from the intestinal bacteria, the M13 metallopeptidase RS7445 was found to show a significant up-regulated expression in response to mucin administration in *L. gasseri* YS1021 and this enzyme was proved to solely decompose mucin into small peptides in vitro. Moreover, we also investigated that whether mucin could be utilized as the carbon source by the strain YS1021. When cultivated in the modified media B+M minus glucose, the strain YS1021 hardly grew, revealing that mucin could not be consumed for supply of the carbon source nutrient. Hence, it’s speculated that mucin probably was utilized as the nitrogen source by *L. gasseri* YS1021. The results might be attributed to the genetic character of the vaginal *L. gasseri* species, which was found to barely express debranching enzymes (sialidase, fucosidase, galactosidase, and sulfatase) for utilization of O-glycan side chains of mucin [12]. Nevertheless, as mucin could be broken down to support bacterial growth, it’s reasonable to assume that some carbohydrate-active enzymes (CAZymes) should be expressed to degrade these glycan side chains attached to the peptide backbone of mucin in *L. gasseri* YS1021. The question that which CAZymes get involved in mucin O-glycans degradation in *L. gasseri* YS1021 is still a mystery and needs further investigation.

Extensive attentions have been focused on mucin O-glycans degradation and corresponding CAZymes such as glycoside hydrolases, sulfatases and esterases in microbiota members [28]. According to the glycoprotein structure of mucin, it’s rational to assume that the surrounding glycan chains should be initially cut off to expose the peptide chain backbone for subsequent proteolytic process. The finding that the purified M13 metallopeptidase RS7445 could directly decompose mucin into peptide fragments in vitro suggested that mucin degradation in *L. gasseri* YS1021 probably was not the case that needs sequential actions of CAZymes and peptidases. Another metalloprotease Amuc_0627 (GenBank No. ACD04464.1) that is capable of degrading mucin 2 (MUC2) was found in the mucin-degrading gut microbe *A. muciniphila*, which belongs to the M60-like or Pfam 13402 family [29]. RS7445 and Amuc_0627 shared a less than 10% similarity in protein sequence, indicating the two mucinases from different protein families are quite different. Moreover, RS7445 also exhibited the proteolytic activity against collagen, which indicated that RS7445 was not selective for mucin. Members of the M13 metalloprotease family are known to cleave a variety of peptide substrates. But it’s not clear whether RS7445 has a broader protease/glycoprotein-associated protease with relatively general substrate scope, which is worth in-depth study. Even though RS7445 is not specifically adapted to mucin, the mucin-degrading enzyme RS7445 will benefit the host *Lactobacillus* strain as mucin is the main nutrient source in the female genital tract. Further characterization of RS7445 is indispensable for fully understanding of its role in mucin degradation by *L. gasseri* YS1021.

## 5. Conclusions

The strain *L. gasseri* YS1021 could effectively degrade and utilize mucin. The M13 family metalloprotease RS7445 was confirmed to be involved in mucin degradation, whose over-expression resulted in an enhanced capacity of mucin degradation and utilization for the host strain YS1021. The 74 kDa recombinant protein of RS7445 was successfully expressed and purified from *E. coli* BL21 and it could decompose mucin into hundreds of peptide fragments. The results revealed that the vaginal *L. gasseri* YS1021 can effectively feed on mucin by the involvement of the M13 metallopeptidase RS7445.

## Figures and Tables

**Table 1:** Strains and plasmids used in this study.

Strains or Plasmids	Relevant Features	Source
*L. gasseri* YS1021	wild strain, donor of *RS7445*	Laboratory collection
*L. gasseri* 7445OE	*L. gasseri* YS1021 harboring pSIP7445	This work
*E. coli* TOP10	host strain used for gene cloning	TIANGEN, Beijing, China
*E. coli* BL21 (DE3)	host strain used for protein expression	TIANGEN, Beijing, China
*E. coli* 7445OE	*E. coli* BL21 (DE3) harboring pET7445	This work
pSIP411	vector for gene expression in *L. gasseri*, Ery ^R^	Laboratory collection
pSIP7445	*Nco* I-*Eco*R I digested P1/P2 PCR product of *RS7445* cloned in pSIP411, Ery ^R^	This work
pET-28a	vector for protein expression in *E. coli*, Kana ^R^	Laboratory collection
pET7445	P7/P8 PCR product of *RS7445* cloned in pET-28a, Kana ^R^	This work

Ery ^R^, erythromycin resistance; Kana ^R^, kanamycin resistance.

**Table 2:** Primers used for PCR and RT-qPCR in this work.

Primer	Sequence (5′-3′)
P1 (RS7445-F)	TGGCTTGTAGTTACCTTCATTAAT
P2 (RS7445-R)	TACCATAGTAGAGACCAATGACTTC
P3 (GADPH-F)	CAACGTGTTCCAGTTCCAGATG
P4 (GADPH-R)	CGCTAGAAACGATGTTGTGGTC
P5 (7445-qPCR-F)	AGGCGAAGTCATTGGTCTCTAC
P6 (7445-qPCR-R)	TGTAGAAGACGTAAGCCAGTCG
P7 (7445-YYBD-F)	CTTTAAGAAGGAGATATACCATGAACCGTTTCAAGATCCGTG
P8 (7445-YYBD-R)	GGTGGTGGTGGTGGTGCTCGTTAGTGGTGGTGGTGGTGGTG
P9 (pET-28a-F)	CGAGCACCACCACCACCA
P10 (pET-28a-R)	GGTATATCTCCTTCTTAAAGTTAAACAAAAT

**Table 3:** Possible peptidase genes involved in mucin degradation in *L. gasseri* YS1021.

Peptidase Gene	Classification	Expression Level by Transcriptome Analysis	Expression Level by RT-qPCR Assay
*RS2420*	Metallopeptidase, Peptidase family M13	1.91	1.38
*RS5645*	Aminopeptidase, Peptidase C1-like family	1.91	0.47
*RS6565*	Peptidase M13, Peptidase family M13	1.82	1.32
*RS6040*	Dipeptidase, Peptidase family C69	1.66	1.52
*RS7445*	Metallopeptidase, Peptidase family M13	3.72	6.04

## Data Availability

The data that support the findings of this study are available on request from the corresponding authors.

## Supplementary Materials

The following supporting information can be downloaded at: https://www.mdpi.com/article/10.3390/microorganisms14051136/s1, Table S1: LC-MS/MS analysis of enzymolysis products of mucin by RS7445.
